# Therapeutic potential and mechanisms of plant metabolites in endometritis: a comprehensive review

**DOI:** 10.3389/fphar.2026.1818049

**Published:** 2026-05-07

**Authors:** Qingcan Guan, Hongxia Gao, Xiaoyan Pan

**Affiliations:** 1 Center for Reproductive Medicine, Jilin Medical University, Jilin, China; 2 School of Medical Technology, Beihua University, Jilin, China

**Keywords:** endometritis, inflammation, plant metabolites, signaling pathways, therapeutic targets

## Abstract

Endometritis is a common gynecological disorder predominantly affecting women of reproductive age. This condition is clinically characterized by symptoms including abdominal pain and irregular bleeding, often leads to impaired fertility, and is primarily driven by infection, injury, or endocrine disturbances. Conventional antibiotic therapy faces considerable challenges, including side effects and the rising threat of antimicrobial resistance, necessitating the exploration of safer and more effective treatment alternatives. Notably, plant metabolites have emerged as promising candidates for endometritis treatment, offering broad natural sources, low toxicity, and multi-target actions on the endometrium. These metabolites exert therapeutic effects by modulating key mechanistic pathways, including the suppression of pro-inflammatory signaling (e.g., NF-κB and MAPK pathways), inhibition of the NLRP3 inflammasome, activation of the antioxidant Nrf2 pathway, and regulation of various cell death modalities such as apoptosis, pyroptosis, and ferroptosis. Additionally, they contribute to endometrial restoration by strengthening the epithelial barrier, modulating hormonal balance, and inhibiting fibrosis. This review systematically categorizes plant metabolites with demonstrated efficacy against endometritis, elucidates their underlying mechanisms, and summarizes their current applications. Finally, we critically discuss the existing limitations of the field and outline future research priorities. Our findings may provide a theoretical and experimental foundation to facilitate the clinical translation and application of these promising plant metabolites.

## 1 Introduction

Endometritis, an inflammatory condition of the endometrial lining primarily affecting women of reproductive age, represents a significant clinical challenge in reproductive medicine and is a well-recognized cause of pelvic pain, menstrual dysfunction, and impaired fertility (Kuroda et al., 2022). The condition can be classified into acute and chronic forms, with chronic endometritis particularly insidious due to its often asymptomatic or mildly symptomatic presentation, yet it affects an estimated 10%–40% of women with unexplained infertility and recurrent implantation failure (Moreno et al., 2018; Cicinelli et al., 2019). Its management has long relied on antibiotic therapy. However, the efficacy of this standard approach is increasingly compromised by antimicrobial resistance, while its focus on pathogen eradication often fails to address the concomitant inflammatory tissue damage and impaired repair that underlie persistent symptoms and fertility deficits (Huang et al., 2020; Song et al., 2021). This clear therapeutic gap underscores the urgent need for novel strategies capable of simultaneously controlling infection, resolving inflammation, and facilitating functional endometrial recovery.

In this context, plant metabolites have emerged as a promising frontier. These natural metabolites offer a compelling multi-target pharmacological profile, exhibiting not only anti-inflammatory and antimicrobial properties but also the demonstrated potential to enhance cell survival, restore barrier integrity, and inhibit pathological fibrosis (Yang D. et al., 2022; Chen et al., 2023). Several specific metabolites have shown particular promise in the context of endometritis. For example, resveratrol, a polyphenol found in *Vitis vinifera* L. and *Reynoutria japonica* Houtt. (syn. *Polygonum cuspidatum* Siebold and Zucc.), has been demonstrated to attenuate lipopolysaccharide (LPS)-induced endometrial inflammation by inhibiting the nuclear factor kappa-B (NF-κB) and SIRT1 signaling pathways, while also reducing oxidative stress and regulating serum hormone levels (Han et al., 2021). Baicalin, a flavonoid isolated from *Scutellaria baicalensis* Georgi, exhibits potent anti-inflammatory effects in endometritis models by suppressing Toll-like receptor 4 (TLR4)-mediated inflammatory responses and promoting the repair of the endometrial barrier (Yang D. et al., 2022). Additionally, curcumin, the active metabolite of *Curcuma longa* L. has been shown to alleviate endometrial oxidative stress and fibrosis by modulating the nuclear factor erythroid 2-related factor 2 (Nrf2) antioxidant pathway and inhibiting TGF-β1/Smad signaling, thereby preserving endometrial structure and function (Liu M. et al., 2023; Li et al., 2026). These metabolites, among others, represent a growing body of evidence supporting the therapeutic potential of plant metabolites in addressing the complex pathophysiology of endometritis. Therefore, further investigation into their mechanisms of action and clinical applicability is warranted.

Accordingly, this review aims to provide a comprehensive and critical synthesis of current knowledge on plant metabolites against endometritis. We will systematically categorize these metabolites, detail their specific mechanisms of action, and integrate findings from preclinical models. Furthermore, we will discuss existing translational challenges and future research directions. It is hoped that this work will consolidate the existing evidence and provide a robust foundation to stimulate further research and accelerate the development of these promising natural metabolites for endometritis management.

## 2 Literature search methodology

The evidence presented in this review was synthesized through a structured search of the scientific literature. We systematically queried electronic databases, including PubMed and the CNKI (China National Knowledge Infrastructure), covering publications from the past decade. The search strategy employed a combination of MeSH (Medical Subject Headings) terms and keywords, such as “*Escherichia coli*,” “*Staphylococcus aureus*,” “pharmacological action,” “botanical drug,” “traditional Chinese medicine,” “bioactive,” “plant,” “metabolites,” and “endometritis.” Additional reference sources included the Pharmacopoeia of the People’s Republic of China. The review focused on studies that investigated plant metabolites in the context of endometritis (including pelvic inflammatory disease).

Inclusion criteria were: (1) original research articles or peer-reviewed reviews (in English or Chinese); (2) studies involving clearly identified and sourced plant metabolites; (3) studies evaluating the effects of plant metabolites on endometritis or related inflammatory conditions; (4) studies reporting mechanistic data on signaling pathways, oxidative stress, cell death, or tissue repair. Exclusion criteria were: (1) conference abstracts, case reports, editorials; (2) studies not reporting original data (for research articles); (3) articles focusing on synthetic drugs only without natural metabolites.

The nomenclature of botanical drugs was verified against the Medicinal Plant Names Services (MPNS, http://mpns.kew.org/mpns-portal/). The metabolites discussed were confirmed to be obtained via standard extraction and preparation methods and clearly identified through mass spectrometry. A summary of the major metabolites reviewed is provided in Table 1. The literature search and taxonomic verification followed the best practice guidelines for ethnopharmacological research (Heinrich et al., 2020). The ConPhyMP guidelines (Heinrich et al., 2022) were consulted for the chemical characterization of plant metabolites (see Supplementary Material for completed GA checklists).

**TABLE 1:** Plant metabolites for the treatment of endometritis.

Classification	Plant metabolite (English name)	Plant metabolite (Chinese name)	Source plant (English)	Source plant (Chinese)	Family	Drug name (Pharmacopoeia)	Medicinal part (English)	Medicinal part (Chinese)	References
Organic Acids									
​	Cichoric Acid	菊苣酸	*Echinacea purpurea* (L.) Moench	紫锥菊	Asteraceae	Echinaceae herba	Whole plant	全草	Yang M. et al. (2022)
​	​	​	*Cichorium intybus* L.	菊苣	Asteraceae	Cichorii herba et radix	Whole plant and root	全草,根	Janda et al. (2021)
​	Ferulic Acid	阿魏酸	*Angelica sinensis* (Oliv.) Diels	当归	Apiaceae	Angelicae sinensis radix	Root	根	de Oliveira Silva and Batista (2017)
​	​	​	*Conioselinum anthriscoides* 'Chuanxiong'	川芎	Apiaceae	Chuanxiong rhizoma	Rhizome	根茎	de Oliveira Silva and Batista (2017)
​	​	​	*Ferula assa-foetida* L.	阿魏	Apiaceae	—	Resin	树脂	de Oliveira Silva and Batista (2017)
​	Chlorogenic Acid	绿原酸	*Lonicera japonica* Thunb.	金银花	Caprifoliaceae	Lonicerae japonicae flos	Flower bud	花蕾	Yuan et al. (2025)
​	​	​	*Eucommia ulmoides* Oliv.	杜仲	Eucommiaceae	Eucommiae cortex	Bark	树皮	Yuan et al. (2025)
Alkaloids									
​	Nuciferine	荷叶碱	*Nelumbo nucifera* Gaertn.	莲	Nelumbonaceae	Nelumbinis folium	Lotus leaf	荷叶	Liu et al. (2025a)
​	Leonurine	益母草碱	*Leonurus japonicas* Houtt.	益母草	Lamiaceae	Leonuri herba	Whole plant	全草	Liu et al. (2024)
​	Berberine	小檗碱	*Coptis chinensis* Franch.	黄连	Ranunculaceae	Coptidis rhizoma	Rhizome	根茎	Fan et al. (2025)
​	​	​	*Phellodendron chinense* C.K.Schneid.	黄柏	Rutaceae	Phellodendri chinensis cortex	Bark	树皮	Fan et al. (2025)
​	Matrine	苦参碱	*Sophora flavescens* Aiton	苦参	Fabaceae	Sophorae flavescentis radix	Root	根	Xu et al. (2023)
​	​	​	*Euchresta japonica* Hook.f. ex Regel	山豆根	Fabaceae	Sophorae tonkinensis radix et rhizoma	Root and rhizome	根及根茎	Xu et al. (2025)
​	Piperine	胡椒碱	*Piper nigrum* L.	胡椒	Piperaceae	Piperis nigri fructus	Nearly mature or mature fruit	近成熟或成熟果实	Haq et al. (2021)
​	​	​	*Piper longum* L.	荜茇	Piperaceae	Piperis longi fructus	Nearly mature or mature fruit spike	近成熟或成熟果穗	Pan et al. (2025)
Saponins									
​	Astragaloside IV	黄芪甲苷IV	*Astragalus mongholicus* Bunge	黄芪	Fabaceae	Astragali radix	Root	根	Zhang J. et al. (2020)
​	Ginsenoside Rb1	人参皂苷Rb1	*Panax ginseng* C.A.Mey.	人参	Araliaceae	Ginseng radix et rhizoma	Root and rhizome	根和根茎	Lu et al. (2018)
​	​	​	*Panax quinquefolius* L.	西洋参	Araliaceae	Panacis quinquefolii radix	Root	根	Jin et al. (2026)
​	Ginsenoside Rg1	人参皂苷Rg1	*Panax ginseng* C.A.Mey.	人参	Araliaceae	Ginseng radix et rhizoma	Root and rhizome	根和根茎	Lu et al. (2018)
​	​	​	*Panax quinquefolius* L.	西洋参	Araliaceae	Panacis quinquefolii radix	Root	根	Jin et al. (2026)
​	Timosaponin AIII	知母皂苷AIII	*Anemarrhena asphodeloides* Bunge	知母	Asparagaceae	Anemarrhenae rhizoma	Rhizome	根茎	Han et al. (2018)
​	Saikosaponin A	柴胡皂苷A	*Bupleurum chinense* DC.	柴胡	Apiaceae	Bupleuri radix	Root	根	Lim et al. (2021)
Polyphenols									
​	Resveratrol	白藜芦醇	*Vitis vinifera* L.	葡萄	Vitaceae	Vitis viniferae fructus	Fruit	果实	Kiskova et al. (2020)
​	​	​	*Reynoutria japonica* Houtt.	虎杖	Polygonaceae	Polygoni cuspidati rhizoma et radix	Rhizome and root	根茎和根	Liu et al. (2025b)
​	Curcumin	姜黄素	*Curcuma longa* L.	姜黄	Zingiberaceae	Curcumae longae rhizoma	Rhizome	根茎	Zagorska et al. (2023)
​	Punicalagin	安石榴苷	*Punica granatum* L.	石榴	Lythraceae	Granati pericarpium	Pericarp, Leaf	果皮,叶	El-Aguel et al. (2022)
​	Rosmarinic Acid	迷迭香酸	*Salvia Rosmarinus* Spenn.	迷迭香	Lamiaceae	Rosmarini folium	Whole plant	全草	Kola et al. (2024)
​	​	​	*Perilla frutescens* (L.) Britton	紫苏	Lamiaceae	Perillae folium	Leaf	叶	Lee et al. (2023)
​	Polydatin	虎杖苷	*Reynoutria japonica* Houtt.	虎杖	Polygonaceae	Polygoni cuspidati rhizoma et radix	Rhizome and root	根茎和根	Luo et al. (2022)
​	Magnolol	厚朴酚	*Magnolia officinalis* Rehder & E.H.Wilson	厚朴	Magnoliaceae	Magnoliae officinalis cortex	Dry bark, root bark, and branch bark	干皮,根皮及枝皮	Zhang et al. (2024)
​	Honokiol	和厚朴酚	*Magnolia officinalis* Rehder & E.H.Wilson	厚朴	Magnoliaceae	Magnoliae officinalis cortex	Dry bark, root bark, and branch bark	干皮,根皮及枝皮	Zhang et al. (2024)
​	Hydroxytyrosol	羟基酪醇	*Olea europaea* L.	油橄榄	Oleaceae	Oleae europaeae folium	Leaf, Fruit	叶,果实	Bertelli et al. (2020)
Terpenoids									
​	Citral	柠檬醛	*Cymbopogon citratus* (DC.) Stapf	柠檬草	Poaceae	Cymbopogonis citrati herba	Whole plant	全草	Hu et al. (2025)
​	​	​	*Litsea cubeba* (Lour.) Pers.	山苍子	Lauraceae	Litseae cubebae fructus	Fruit	果实	Hu et al. (2025)
​	Oridonin	冬凌草甲素	*Isodon rubescens* (Hemsl.) H.Hara	冬凌草	Lamiaceae	Isodontis rubescentis herba	Whole plant	全草	Zhang Y. et al. (2020)
​	Andrographolide	穿心莲内酯	*Andrographis paniculata* (Burm.f.) Wall. ex Nees	穿心莲	Acanthaceae	Andrographis herba	Stem, Leaf	茎,叶	Zeng B. et al. (2022)
​	Catalpol	梓醇	*Rehmannia glutinosa* (Gaertn.) Libosch. ex DC.	地黄	Orobanchaceae	Rehmanniae radix	Tuberous root	块根	Ma et al. (2024)
​	Aucubin	桃叶珊瑚苷	*Eucommia ulmoides* Oliv.	杜仲	Eucommiaceae	Eucommiae cortex	Bark	树皮	Kartini et al. (2023)
​	​	​	*Plantago asiatica* L.	车前草	Plantaginaceae	Plantaginis herba	Whole plant	全草	Kartini et al. (2023)
​	Thymol	百里香酚	*Thymus vulgaris* L.	百里香	Lamiaceae	Thymi herba	Whole plant	全草	Salehi et al. (2018)
Flavonoids									
​	Puerarin	葛根素	*Pueraria montana* var. *lobate* (Willd.) Maesen & S.M.Almeida ex Sanjappa & Predeep	葛根	Fabaceae	Puerariae lobatae radix	Root	根	Zhou et al. (2014)
​	Engeletin	黄杞苷	*Engelhardia roxburghiana* Wall.	黄杞	Juglandaceae	—	Bark, Leaf	树皮,叶	Wei et al. (2020)
​	Baicalin	黄芩苷	*Scutellaria baicalensis* Georgi	黄芩	Lamiaceae	Scutellariae radix	Root	根	Meng et al. (2025)
​	Alpinetin	山姜素	*Alpinia officinarum* Hance	山姜	Zingiberaceae	Alpiniae officinarum rhizoma	Rhizome	根茎	Zhao et al. (2022)
​	Luteolin	木犀草素	*Taraxacum officinale* F.H.Wigg.	蒲公英	Asteraceae	Taraxaci herba	Flower	花	Jedrejek and Pawelec (2024)
​	Apigenin	芹菜素	*Apium graveolens* L.	芹菜	Apiaceae	Apii graveolentis herba	Leaf and stem	叶,茎	Charriere et al. (2024)
​	​	​	*Lonicera japonica* Thunb.	金银花	Caprifoliaceae	Lonicerae japonicae flos	Whole plant	全草	Charriere et al. (2024)
​	Icariin	淫羊藿苷	*Epimedium brevicornu* Maxim.	淫羊藿	Berberidaceae	Epimedii folium	Leaf, Stem	叶,茎	Bi et al. (2022)
​	Cyanidin-3-O-glucoside	矢车菊素-3-O-葡萄糖苷	*Vaccinium uliginosum* L.	蓝莓	Ericaceae	—	Fruit	果实	Olivas-Aguirre et al. (2016)
​	Naringin	柚皮苷	*Citrus × limon* (L.) Osbeck	柚子	Rutaceae	Citri grandis fructus	Fruit peel	果实的皮	Shangguan et al. (2023)
​	Fisetin	漆黄素	*Toxicodendron vernicifluum* (Stokes) Lincz.	漆树	Anacardiaceae	—	Bark, Leaf	树皮,叶	Li et al. (2021)
​	Hydroxysafflor Yellow A	羟基红花黄色素A	*Carthamus tinctorius* L.	红花	Asteraceae	Carthami flos	Flower	花	Wang et al. (2024)
​	Tanshinone IIA	丹参酮IIA	*Salvia miltiorrhiza* Bunge	丹参	Lamiaceae	Salviae miltiorrhizae radix et rhizoma	Root and rhizome	根和根茎	Li et al. (2018)

## 3 Epidemiology and current therapeutic landscape of endometritis

### 3.1 Clinical epidemiology of endometritis

Endometritis is defined as an infectious inflammation of the endometrium. Based on disease duration, it is categorized into acute and chronic forms. Acute endometritis, often secondary to postpartum or post-procedural events, is characterized by infection lasting ≤30 days (Ravel et al., 2021; Singh and Sethi, 2022). Chronic endometritis involves persistent inflammation (>30 days) and is a recognized contributor to recurrent miscarriage and infertility (Ravel et al., 2021). The condition is prevalent, affecting an estimated 8%–15% of women of reproductive age (Cicinelli et al., 2018). Its incidence increases in populations with reproductive dysfunction: approximately 51.7% in women with unexplained infertility (Gu et al., 2023), 40.2% in those experiencing recurrent implantation failure (Amrani et al., 2025), and ranging from 9.3% to 67.6% in women with a history of recurrent pregnancy loss (Murtinger et al., 2022).

The principal risk factors associated with endometritis include obstetric events (postpartum infection, cesarean delivery, and retained products of conception), gynecological interventions (hysteroscopy, intrauterine device insertion, endometrial biopsy, and curettage), infectious transmissions (sexually transmitted infections, bacterial vaginosis, and pelvic inflammatory disease), as well as immunocompromised status and intrauterine adhesions (Singh and Sethi, 2022; Taylor et al., 2023).

Clinically, acute cases typically present with overt symptoms such as fever, severe lower abdominal pain, and purulent discharge. Chronic endometritis, however, frequently manifests with subtler signs including irregular uterine bleeding, mild chronic pelvic pain, and subfertility, often leading to underdiagnosis (Taylor et al., 2023).

### 3.2 Etiology and pathogenesis

The etiology of endometritis is predominantly infectious, with pathogens implicated in 80%–90% of cases (Ravaioli et al., 2022). Common routes of infection include ascending cervical invasion, hematogenous spread, and iatrogenic introduction. *Escherichia coli* is a major pathogen in acute settings, accounting for over 30% of infections (Salmanov et al., 2020). Other significant bacteria include *Streptococcus* spp., *Staphylococcus* spp., *Klebsiella spp.*, and sexually transmitted agents such as *Chlamydia trachomatis* and *Neisseria gonorrhoeae* (He et al., 2025). Chronic endometritis often involves a more complex, polymicrobial profile and biofilm formation, which contribute to therapeutic recalcitrance (Swidsinski et al., 2013). In veterinary medicine, *Trueperella pyogenes* is a key pathogen, with virulence factors such as hemolysin playing a crucial role (Li D. et al., 2025).

The core pathogenesis involves pathogen recognition by endometrial epithelial cells via pattern recognition receptors (e.g., TLR2 and TLR4), triggering intracellular signaling cascades such as NF-κB. This leads to the robust production of pro-inflammatory cytokines (e.g., TNF-α, IL-1β, IL-6) and chemokines, recruiting innate immune cells such as neutrophils and macrophages to the site. Histologically, this phase is characterized by neutrophil infiltration and micro-abscess formation (Ravel et al., 2021; Singh and Sethi, 2022). While this response aims to clear the infection, excessive or persistent inflammation results in tissue damage, including congestion, edema, and glandular disruption. If unresolved, this progresses to a chronic phase characterized by lymphocyte and plasma cell infiltration, aberrant tissue repair, collagen deposition, and fibrosis. These pathological changes ultimately degrade endometrial receptivity and function, underpinning the associated fertility deficits (Yan et al., 2025).

### 3.3 Current treatment modalities

#### 3.3.1 Antibiotic therapy

Antibiotics constitute the first-line treatment for bacterial endometritis. For acute cases, broad-spectrum regimens such as ceftriaxone combined with metronidazole are commonly prescribed for 1–2 weeks, achieving clinical remission rates of 80%–90% (Wiesenfeld et al., 2021). Chronic endometritis often necessitates prolonged courses of 2–4 weeks (Zhou et al., 2016). However, this approach faces significant challenges in chronic disease, where reported resistance rates can reach 30%–40%. Recurrence is common (25%–35%), particularly with infections involving *Mycoplasma* and anaerobes, and the prevalence of multidrug-resistant organisms is rising (Kitaya et al., 2022). Furthermore, protracted antibiotic use disrupts the balance of the reproductive tract microbiota, increasing the risk of secondary infections (Yan et al., 2025). Critically, even after successful microbial clearance, approximately 30%–40% of patients may continue to experience embryo implantation failure, indicating that underlying endometrial damage and dysfunction persist despite resolution of the active infection (Huang et al., 2020). These limitations highlight the necessity for adjunctive strategies that not only combat infection but also promote tissue repair and functional recovery.

#### 3.3.2 Integrated traditional Chinese and western medicine

Given the limitations of antibiotics, particularly in chronic and resistant cases, integrating Traditional Chinese Medicine (TCM) with conventional therapy has emerged as a promising approach. The combination of certain compound TCM formulations with antibiotics can reverse bacterial drug resistance and exert synergistic therapeutic effects against drug-resistant infections (Qiu et al., 2025). Several TCM formulas have demonstrated clinical benefits in endometritis management. In the chronic phase or as postoperative adjunctive therapy, formulas with properties of “clearing heat, removing toxins, and activating blood circulation” have shown particular promise. Zaogong Erteng Decoction significantly suppresses the expression of inflammatory factors (e.g., IL-6, TNF-α) and improves uterine microcirculation (Li S. et al., 2025). Kangfuxiaoyan Suppository reduces the recurrence rate of endometritis by 10%–15% compared to antibiotic therapy alone (Zhang et al., 2022; Zhang and Liu, 2025). Baogong Decoction exhibits efficacy against *E. coli*-induced endometritis, potentially through modulating the gut microbiota and related metabolites (Ding et al., 2022). Fuke Qianjin Capsule, a widely used TCM preparation for endometritis, alleviates LPS-induced inflammation by inhibiting the TLR4/NF-κB pathway (Xiong et al., 2025).

These formulations thus provide adjunctive anti-inflammatory benefits while contributing to endometrial protection, addressing the shortcomings of purely antimicrobial therapy. The therapeutic effects of these complex formulas are attributable to their metabolites, which are discussed in detail in the following section.

#### 3.3.3 Treatment with plant metabolites

Beyond their role within complex botanical drug formulations, isolated plant metabolites themselves represent a promising and distinct therapeutic strategy for endometritis. These metabolites possess diverse biological activities, including anti-inflammatory, antimicrobial, and immunomodulatory effects. When used as adjuvants alongside antibiotics, they can achieve the goal of “enhancing efficacy and reducing toxicity” (Khan et al., 2024). Some metabolites enhance antibiotic efficacy while mitigating their adverse effects. For example, baicalein, when combined with doxycycline, can reduce resistance rates in *Chlamydia* infections while mitigating antibiotic-induced gut dysbiosis (Wang et al., 2023). Diammonium glycyrrhizinate, a derivative of glycyrrhizin from licorice root, exerts anti-inflammatory effects, inhibits the secretion of inflammatory cytokines, improves endometrial receptivity. Notably, it shows no adverse effects on embryonic development, making it a suitable adjunctive therapy for women who are planning pregnancy or are already pregnant (Wang et al., 2017). Other metabolites primarily contribute to endometrial recovery—an outcome unattainable with antibiotics alone. Metabolites derived from TCM botanical drugs illustrate this potential: Total flavonoids of safflower (*Carthamus tinctorius* L.) can inhibit inflammation by regulating the estrogen receptor α/phosphatidylinositol 3-kinase (PI3K)/protein kinase B (AKT) pathway while reducing excessive apoptosis of uterine tissue cells (Chen et al., 2023). Danshen (*Salvia miltiorrhiza* Bunge)-derived metabolites including tanshinone IIA, cryptotanshinone, and salvianolic acids A/B demonstrate anti-inflammatory and energy balance actions, improving uterine blood flow and coagulation function to mitigate uterine inflammation (Tian et al., 2024).

These metabolites thus enhance local blood circulation and facilitate the restoration of endometrial function. To facilitate a deeper understanding and effective utilization of these metabolites, Tables 2, 3 summarize the therapeutic effects and mechanisms of key plant metabolites in the context of endometritis.

**TABLE 2:** Therapeutic effects of plant metabolites in endometritis: *in vivo* studies.

Plant metabolites	Experimental subject	Model induction	Treatment regimen	Therapeutic effects	References
Cichoric acid	C57BL/6 mice; Nrf2-knockout mice (C57BL/6J background)	Intrauterine infusion of 100 μL (1 mg/mL) LPS, 24 h	i.p. injection of 40 mg/kg cichoric acid 1 h before LPS, total 25 h	Reduced endometrial epithelial exfoliation and inflammatory cell infiltration; lowered TNF-α, IL-6, and IL-1β levels; inhibited ferroptosis; decreased MPO activity; suppressed NF-κB activation; upregulated Nrf2 and HO-1 expression	Wang et al. (2022)
Chlorogenic acid	BALB/c mice	Intrauterine perfusion with a mixture of LPS and pyolysin, 50 μL per infusion, 5 times	i.p. injection of 50 mg/kg chlorogenic acid daily for 3 days after modeling	Alleviated uterine swelling and purulent discharge; reduced uterine index, MPO activity, and TNF-α, IL-1β, IL-6 levels; inhibited NF-κB signaling; activated Keap1/Nrf2 signaling; restored blood leukocyte, lymphocyte, and neutrophil counts	Gao et al. (2021a)
Nuciferine	C57BL/6 mice	Intrauterine infusion of 50 μL (1 mg/mL) LPS per horn, 24 h	Oral gavage of 30 mg/kg nuciferine daily for 5 days before LPS.	Reduced uterine congestion and inflammatory cell infiltration; lowered MPO activity and TNF-α, IL-6, IL-1β levels; inhibited ferroptosis; suppressed MyD88/NF-κB and MAPK pathways; regulated AMPKα/mTOR/HIF-1α signaling; improved pregnancy rate and litter size	Kim et al. (2022)
Leonurine	C57BL/6J mice	Bilateral intrauterine horn infusion of 50 μL (1 mg/mL) LPS, 24 h	i.p. injection of 30 mg/kg of Leonurine 1 h before LPS, followed by injections every 6 h for a total of 4 doses	Reduced TNF-α, IL-6, and IL-1β levels; regulated gene expression in JAK-STAT/PI3K-Akt pathway (e.g., Prlr, Socs2, Col1a1, Akt1); inhibited excessive inflammation and fibrosis; modulated PPAR pathway genes (e.g., Cyp27a1, Hmgcs1, Scd2), improving local lipid metabolism microenvironment and promoting uterine repair	Shao et al. (2024)
Berberine hydrochloride	BALB/c mice	Intrauterine infusion of 20 μL (2.5 mg/mL) LPS, 24 h	i.p. injection of 10 mg/kg Berberine 1 h before and 12 h after LPS.	Reduced inflammatory cell infiltration; decreased MPO activity, NO levels, and TNF-α, IL-1β levels; activated Keap1/Nrf2 signaling pathway, upregulating HO-1, NQO1 expression	Fu et al. (2015)
Matrine	BALB/c mice	Intrauterine injection of 5 mg/kg S. aureus LTA, 24 h	i.p. injection of 100 mg/kg Matrine every 8 h, 3 times after modeling	Reduced uterine congestion, hemorrhage, and inflammatory cell infiltration; lowered TNF-α, IL-1β expression in uterine tissue; inhibited TLR2 expression and NF-κB activation	Jiang et al. (2019)
Piperine	BALB/c mice	Intrauterine injection of 100 μL *S. aureus* suspension per horn, 24 h	i.p. injection of 100 mg/kg Piperine every 6 h after modeling, 3 times	Alleviated uterine inflammatory injury; decreased TNF-α, IL-1β, IL-6 expression, increased IL-10; lowered TLR-2 and TLR-4 expression; inhibited phosphorylation of I-κB, p65, p38, ERK, and JNK.	Zhai et al. (2016)
Astragaloside IV	BALB/c mice	Intrauterine infusion of 25 μL (2.5 mg/mL) LPS, 24 h	Oral gavage of 0.01 mg/g Astragaloside IV daily for 6 days before LPS.	Reduced endometrial epithelial exfoliation and inflammatory cell infiltration; decreased uterine MPO activity, NO concentration, and IL-1β, TNF-α levels; inhibited TLR4 expression and NF-κB, p38, JNK pathway activation	Wang et al. (2019)
Ginsenoside Rb1	BALB/c mice	Intrauterine infusion of 50 μL (1 mg/mL) LPS, 24 h	i.p. injection of 50 mg/kg ginsenoside Rb1 every 8 h after modeling, 3 times	Alleviated uterine congestion, hemorrhage, and inflammatory cell infiltration; reduced uterine MPO activity and uterine index (edema); decreased TNF-α, IL-1β, IL-6 expression, increased IL-10	Shaukat et al. (2021)
Ginsenoside Rg1	ICR mice	Intrauterine infusion of 50 μL (1 mg/mL) LPS, every 2 days for 6 days	Concurrent i.p. injection of 100 mg/kg ginsenoside Rg1 daily for 6 days during modeling	Inhibited EMT; reduced ROS generation, inhibiting NLRP3 inflammasome activation; alleviated endometrial fibrosis in mice; mitigated oxidative stress (reduced MDA, increased SOD and CAT activity)	Song et al. (2023)
Timosaponin AIII	BALB/c mice	Intrauterine injection of *E. coli* (1 × 106 CFU/mL, 50 μL per horn), 24 h	Oral gavage of 20 mg/kg timosaponin AIII daily for 5 days before modeling	Reduced uterine inflammatory cell infiltration, hemorrhage, edema; decreased uterine MPO activity and TNF-α, IL-1β, IL-6 levels; restored tight junction protein (ZO-1, Occludin, Claudin-1) expression; inhibited TLR4/NF-κB pathway and NLRP3 inflammasome activation; modulated uterine microbiota structure	Jiang and Xu (2024)
Saikosaponin A	C57BL/6 mice	Intrauterine injection of 20 μL (2.5 mg/mL) LPS, 24 h	i.p. injection of 20 mg/kg saikosaponin A 1 h before LPS, total 25 h	Reduced uterine inflammatory cell infiltration and epithelial exfoliation; decreased uterine MPO activity and TNF-α, IL-1β, IL-6 levels; inhibited NF-κB pathway activation; activated Nrf2 signaling pathway	Wei et al. (2016)
Resveratrol	Sprague Dawley rats	Intrauterine injection of *E. coli* (1 × 105 CFU/rat, 50 μL), 16 h	Intramuscular injection of 30 mg/kg Resveratrol daily for 14 days after modeling	Increased total antioxidant status in uterine tissue, decreased oxidative stress index; reduced serum IL-6, CINC-3, LIX levels; decreased uterine congestion, edema; lowered serum estradiol, raised progesterone, modulated ER and PR expression; reduced intrauterine bacterial load; increased rate of estrous cycle recovery	Han et al. (2021)
Rosmarinic acid	BALB/c mice	Intrauterine horn injection of 2.5 mg/kg LPS, 24 h	Intrauterine injection of 10 mg/kg rosmarinic acid combined with exosomes 24 h after modeling	Inhibited MPO activity; significantly reduced gene/protein expression of TLR4, NLRP3, and pyroptosis factor GSDM-D; decreased levels of inflammatory cytokines (IL-1β, IL-18, TNF-α)	Taravat et al. (2023)
Polydatin	BALB/c mice	Intrauterine perfusion of 50 μL (1 mg/mL) LPS, 24 h	i.p. injection of 80 mg/kg Polydatin before LPS, total 24 h	Alleviated uterine injury, reduced MPO activity, decreased TNF-α, IL-1β, IL-6 levels; inhibited NF-κB activation; enhanced Nrf2 and HO-1 expression	Li et al. (2019)
Oridonin	BALB/c mice	Bilateral intrauterine infusion of 50 μL (1 mg/mL) LPS, 24 h	i.p. injection of 40 mg/kg Oridonin every 8 h after modeling, 3 times	Alleviated uterine pathological changes; decreased MPO activity; reduced TNF-α, IL-1β, IL-6 expression	Zhou et al. (2019)
Catalpol	Kunming mice	Intrauterine injection of 1 mg/kg LPS, 24 h	i.p. injection of 100 mg/kg Catalpol for 24 h after modeling	Reduced TNF-α, IL-1β, IL-6, CXCL5, and CXCL8 expression; decreased MPO activity; alleviated uterine pathological damage	Zhang et al. (2019)
Thymol	BALB/c mice	Intrauterine perfusion of 50 μL (1 mg/mL) LPS, 24 h	i.p. injection of 40 mg/kg Thymol every 6 h after modeling, 3 times	Alleviated uterine pathological damage; reduced MPO activity; decreased TNF-α, IL-1β, iNOS, and COX-2 expression	Wu et al. (2017)
Puerarin	BALB/c mice	Intrauterine horn injection of 1 × 107 CFU/100 μL S. aureus	Daily i.p. injection of 100 mg/kg puerarin for 7 consecutive days after modeling	Alleviated uterine pathological damage; reduced MPO activity; decreased TNF-α, IL-1β, IL-6 expression; inhibited ferroptosis; suppressed expression of P2X7, NLRP3, ASC, caspase-1, NF-κB p-p65, p-IκBα	Yuan et al. (2024)
Engeletin	BALB/c mice	Intrauterine perfusion of 50 μL (1 mg/mL) LPS, 24 h	i.p. injection of 100 mg/kg Engeletin every 6 h after modeling, 3 times	Alleviated uterine pathological changes; reduced MPO activity; decreased TNF-α, IL-1β, IL-6, iNOS, COX-2 expression	Wu et al. (2016)
Baicalin	Kunming mice	Intrauterine perfusion of 20 μL (2.5 mg/kg) LPS, 24 h	Oral gavage of 40 mg/kg Baicalin daily for 5 days before modeling	Alleviated uterine pathological damage; reduced IL-1β, IL-6, and TNF-α levels; upregulated CLDN3 and TJP1 expression, downregulated CLDN2	Yang D. et al. (2022)
Alpinetin	C57BL/6 mice	Intrauterine perfusion of 50 μL (1 mg/mL) LPS, 24 h	i.p. injection of 40 mg/kg Alpinetin 1 h before LPS, total 25 h	Alleviated uterine pathological damage; reduced MPO activity; decreased TNF-α, IL-1β, IL-6 expression; inhibited TLR4 expression and NF-κB activation; upregulated PPAR-γ expression	Liang et al. (2018)
Luteolin	C57BL/6J mice; Nrf2-knockout mice (C57BL/6J)	Intrauterine injection of 50 μL *S. aureus* (1 × 107 CFU/50 μL PBS), 24 h	i.p. injection of 40 mg/kg Luteolin 1 h before modeling, total 25 h	Alleviated uterine pathological damage; reduced MPO activity; decreased TNF-α, IL-1β, IL-6 expression; inhibited ferroptosis; activated Nrf2/HO-1 pathway; suppressed NF-κB pathway	Gao et al. (2024)
Apigenin	C57BL/6 mice	Intrauterine perfusion of 20 μL (2.5 mg/mL) LPS, 24 h	i.p. injection of 40 mg/kg Apigenin 1 h before modeling, total 25 h	Alleviated uterine pathological damage; reduced MPO activity and MDA content; decreased TNF-α, IL-1β expression; inhibited NF-κB activation; upregulated Nrf2 and HO-1 expression	Jiang et al. (2018)
Icariin	Kunming mice	Intrauterine perfusion of 50 μL (1 mg/mL) LPS, 24 h	i.p. injection of 50 mg/kg icariin every 6 h after modeling, 3 times	Alleviated uterine pathological damage; reduced MPO activity and NO concentration; decreased TNF-α, IL-1β, IL-6 levels, increased IL-10; reduced MDA and ROS levels, increased SOD, CAT, Gpx1 activity	Shaukat et al. (2022)
Naringin	Kunming mice	Intrauterine injection of 0.5 mg/kg LPS, 24 h	Oral gavage of 80 mg/kg Naringin for 24 h after modeling	Alleviated uterine pathological damage; reduced MPO activity and IL-6, IL-1β, TNF-α levels	Lu et al. (2022)
Fisetin	BALB/c mice	Intrauterine perfusion of 1 mg/kg LPS, 24 h	Intramuscular injection of 50 mg/kg Fisetin twice (12 h interval) starting 2 h before modeling	Alleviated uterine pathological damage; reduced MPO activity and TNF-α, IL-1β levels	Jiang et al. (2021b)
Hydroxysafflor yellow A	BALB/c mice	Bilateral intrauterine injection of 1 × 108 CFU/100 μL S. aureus suspension	Intrauterine injection of 4 mg/kg hydroxysafflor yellow A 12 h after modeling, 24 h total	Alleviated uterine pathological damage; reduced MPO activity and expression of CD45, CD3, ED-1; decreased TNF-α, IL-1β, IL-6 levels	He et al. (2021)

*Abbreviations:* LPS, lipopolysaccharide; LTA, lipoteichoic acid; i. p., intraperitoneal; MPO, myeloperoxidase; NO, nitric oxide; TNF-α, tumor necrosis factor-α; IL-1β, interleukin-1β; IL-6, interleukin-6; NF-κB, nuclear factor kappa-B; MAPK, mitogen-activated protein kinase; JNK, c-Jun N-terminal kinase; ERK, extracellular signal-regulated kinase; Nrf2, nuclear factor erythroid 2-related factor 2; HO-1, heme oxygenase-1; ROS, reactive oxygen species; MDA, malondialdehyde; SOD, superoxide dismutase; CAT, catalase; GSH, glutathione; EMT, epithelial-mesenchymal transition; NLRP3, NOD-like receptor pyrin domain-containing 3; ASC, apoptosis-associated speck-like protein containing a CARD; ZO-1, zonula occludens-1; CLDN, claudin; TJP1, tight junction protein 1; ER, estrogen receptor; PR, progesterone receptor; COX-2, cyclooxygenase-2; iNOS, inducible nitric oxide synthase; GSDMD, gasdermin D; AMPK, AMP-activated protein kinase; mTOR, mammalian target of rapamycin; HIF-1α, hypoxia-inducible factor 1α; JAK-STAT, Janus kinase-signal transducer and activator of transcription; PI3K-Akt, phosphatidylinositol 3-kinase-protein kinase B; PPAR, peroxisome proliferator-activated receptor; Keap1, kelch-like ECH-associated protein 1; NQO1, NAD(P)H dehydrogenase, quinone 1; TLR2, Toll-like receptor 2; TLR4, Toll-like receptor 4; MyD88, myeloid differentiation primary response 88; I-κB, inhibitor of nuclear factor kappa-B; P2X7, P2X purinergic receptor 7; CXCL, C-X-C motif chemokine ligand; CINC, cytokine-induced neutrophil chemoattractant; LIX, LPS-induced CXC, chemokine; Gpx1, glutathione peroxidase 1; COL1A1, collagen type I alpha 1 chain; CD45, leukocyte common antigen; ED-1, cluster of differentiation 68.

**TABLE 3:** Therapeutic effects of plant metabolites in endometritis: *In Vitro* studies.

Plant metabolites	Experimental subject	Model induction	Treatment regimen	Therapeutic effects	References
Ferulic acid	bEECs	Treatment with 1 μg/mL LPS for 12 h	Pretreatment with 120 μM FA for 4 h before LPS, for a total of 16 h	Reduced bEEC apoptosis; decreased mRNA levels of IL-1β, IL-6, IL-8, TNF-α; inhibited NF-κB and MAPK pathway activation	Yin et al. (2019)
Nuciferine	mEECs	Treatment with 1 μg/mL LPS for 24 h	Pretreatment with 30 μM nuciferine 1 h before LPS, total 25 h	Inhibited inflammatory factor release and NF-κB activation	Kim et al. (2022)
Berberine	bEECs	Treatment with 10 μg/mL LPS for 24 h	Treatment with 20 μM Berberine hydrochloride for 24 h after LPS.	Downregulated CRP, TNF-α, IL-1β and IL-6 expression; activated Keap1/Nrf2 signaling, promoted Nrf2 nuclear translocation; inhibited NF-κB pathway activation	Fu et al. (2015), Fu et al. (2021a)
Matrine	bEECs	Co-cultured with LTA for 6 h after pretreatment	Pretreatment with 400 μM Matrine 1 h before LTA, for a total of 7 h	Downregulated TNF-α, IL-1β expression; inhibited TLR2 expression and NF-κB activation	Jiang et al. (2019)
Ginsenoside Rg1	BEND	Treatment with 1 μg/mL LPS for 48 h	Treatment with 20 µM Ginsenoside Rg1 for 48 h	Inhibited EMT; reduced ROS generation and increased GSH content, inhibiting NLRP3 inflammasome activation; downregulated ASC, cleaved caspase-1, and IL-1β	Song et al. (2023)
Curcumin	Primary bubaline endometrial stromal cells	Treatment with 0.1 μg/mL LPS or combined with 1 μg/mL LTA for 24 h	Co-treatment with 30 μM Curcumin and LPS/LTA for 24 h	Inhibited PGE2 production; downregulated mRNA expression of IL-1β, IL-6, IL-8, TNF-α	Ali et al. (2020)
Punicalagin	bEECs	Treatment with 30 μg/mL LPS for 12 h	Pretreatment with 20 μg/mL punicalagin for 2 h before LPS.	Improved cell viability; downregulated mRNA expression of pro-inflammatory IL-1β, IL-6, IL-8, TNF-α; inhibited NF-κB and MAPK pathway activation	Lyu et al. (2017)
Magnolol	Mouse uterine epithelial cells	Treatment with 1 μg/mL LPS for 24 h	Pretreatment with 50 μg/mL Magnolol 1 h before LPS, total 25 h	Inhibited TNF-α, IL-6 production; reduced TLR4 expression; suppressed NF-κB activation and MAPK phosphorylation	Luo et al. (2013)
Honokiol	bEECs	Treatment with 1 μg/mL LPS for 12 h	Pretreatment with 20 μM honokiol 1 h before LPS, total 13 h	Improved cell viability; reduced TNF-α, IL-1β, IL-6 expression; inhibited apoptosis; decreased expression of ER stress-related proteins ATF6 and CHOP.	Chen et al. (2021)
Hydroxytyrosol	bEECs	Treatment with 1 μg/mL LPS for 6 h	Pretreatment with 25 μM hydroxytyrosol 1 h before LPS, total 7 h	Reduced ROS levels; decreased TNF-α, IL-6 expression; activated Nrf2 pathway; restored tight junction protein (Claudin, CDH1, TJP1) expression	Gugliandolo et al. (2020)
Oridonin	mEECs	Treatment with 1 μg/mL LPS for 24 h	Pretreatment with 20 μg/mL Oridonin 1 h before LPS, for a total of 25 h	Downregulated TNF-α, IL-1β, IL-6 expression; inhibited TLR4 expression and NF-κB pathway activation	Zhou et al. (2019)
Andrographolide	bEECs	Treatment with 100 μg/mL LPS for 24 h	Pretreatment with 60 μM Andrographolide 1 h before LPS, total 25 h	Reduced NO and iNOS expression; decreased IL-1β, TNF-α, IL-6 levels; inhibited Keap1 expression; activated Nrf2 and its target genes HO-1, NQO-1	Fu et al. (2021b)
Catalpol	bEECs	Treatment with 1 μg/mL LPS for 12 h	Pretreatment with 1 mmol/L Catalpol 1 h before LPS, for a total of 13 h	Downregulated TNF-α, IL-1β, IL-6, CXCL5, CXCL8 expression; inhibited TLR4 expression and NF-κB p65 phosphorylation	Zhang et al. (2019)
Aucubin	bEECs	Treatment with 1 μg/mL LPS for 3 h	Pretreatment with 50 μM aucubin for 6 h before LPS.	Downregulated TNF-α, IL-1β, IL-6, COX-2, and iNOS expression; decreased apoptosis; inhibited NF-κB p65 and IκB phosphorylation and nuclear translocation; activated Keap1/Nrf2 pathway	Gao et al. (2021b)
Thymol	RAW264.7 cells	Treatment with 1 μg/mL LPS for 3 h	Pretreatment with 40 μg/mL Thymol 1 h before LPS.	Downregulated TNF-α, IL-1β, iNOS, and COX-2 expression; inhibited TLR4 expression and NF-κB activation; reduced ROS production	Wu et al. (2017)
Engeletin	HEK293-mTLR4/mMD2 cells	Treatment with 1 μg/mL LPS for 3 h	Treatment with 100 μmol/L Engeletin for 6–12 h after LPS.	Inhibited the expression of TLR4 downstream MyD88, IRAK1, TRAF6, TAK1; suppressed NF-κB activation and p65 nuclear translocation	Wu et al. (2016)
Baicalin	Goat EECs	Treatment with 2 μg/mL LPS for 12 h	Pretreatment with 10 μg/mL Baicalin for 2 h before LPS.	Downregulated IL-1β, IL-6, and TNF-α levels; upregulated CLDN3 and TJP1 expression, downregulated CLDN2; activated autophagy, promoting redistribution of tight junction proteins	Yang D. et al. (2022)
Naringin	bEECs	Treatment with 1 μg/mL LPS for 24 h	Co-treatment with 100 μM Naringin and LPS for 24 h	Decreased ROS production; inhibited expression of ER stress-related proteins p-PERK, p-IRE1α, ATF6; activated PI3K/AKT pathway; inhibited autophagy	Lu et al. (2022)
Fisetin	BEND	Treatment with 1 μg/mL LPS for 6 h	Addition of 50 μg/mL Fisetin 1 h before LPS, total 7 h	Downregulated TNF-α, IL-1β levels; inhibited TLR4 expression and NF-κB activation; reduced ROS and Nox4 production; activated Nrf2/HO-1 pathway	Jiang et al. (2021b)
Hydroxysafflor yellow A	mEECs	Co-culture of S. aureus and mEECs at a 1:1 ratio for 6 h	Addition of hydroxysafflor yellow A during co-culture for 6 h	Downregulated TNF-α, IL-1β, IL-6 levels; inhibited expression of TLR2 and its downstream MyD88, IRAK1, IRAK4, TRAF6; suppressed NF-κB and MAPK pathway activation	He et al. (2021)
Tanshinone IIA	bEECs	Pretreatment with 1 μg/mL LPS for 24 h, then 2 HU pyolysin for 1 h	Pretreatment with 5 μg/mL tanshinone IIA for 2 h before LPS and pyolysin	Improved cell viability, reduced LDH release and IL-1β, IL-6, IL-8, TNF-α levels; inhibited epithelial-mesenchymal transition; suppressed NF-κB activation and Snail2 expression	Fu et al. (2021b)

Abbreviations: bEECs, bovine endometrial epithelial cells; mEECs, mouse endometrial epithelial cells; BEND, bovine endometrial epithelial cell line; EECs, endometrial epithelial cells; LPS, lipopolysaccharide; LTA, lipoteichoic acid; i. p., intraperitoneal; MPO, myeloperoxidase; NO, nitric oxide; TNF-α, tumor necrosis factor-α; IL-1β, interleukin-1β; IL-6, interleukin-6; NF-κB, nuclear factor kappa-B; MAPK, mitogen-activated protein kinase; JNK, c-Jun N-terminal kinase; ERK, extracellular signal-regulated kinase; Nrf2, nuclear factor erythroid 2-related factor 2; HO-1, heme oxygenase-1; ROS, reactive oxygen species; MDA, malondialdehyde; GSH, glutathione; EMT, epithelial-mesenchymal transition; NLRP3, NOD-like receptor pyrin domain-containing 3; COX-2, cyclooxygenase-2; iNOS, inducible nitric oxide synthase; LDH, lactate dehydrogenase; ATF6, activating transcription factor 6; CHOP, C/EBP, homologous protein; IRE, inositol-requiring enzyme; UPR, unfolded protein response; PERK, protein kinase R-like endoplasmic reticulum kinase; PI3K, phosphatidylinositol 3-kinase; AKT, protein kinase B; Keap1, kelch-like ECH-associated protein 1; NQO1, NAD(P)H dehydrogenase, quinone 1; TLR2, Toll-like receptor 2; TLR4, Toll-like receptor 4; MyD88, myeloid differentiation primary response 88; IRAK, interleukin-1 receptor-associated kinase; TRAF6, TNF, receptor-associated factor 6; TAK1, transforming growth factor-β-activated kinase 1; I-κB, inhibitor of nuclear factor kappa-B; CXCL, C-X-C motif chemokine ligand; CDH1, cadherin 1; CLDN, claudin; TJP1, tight junction protein 1; Nox4, NADPH, oxidase 4; GSDMD, gasdermin D; ASC, apoptosis-associated speck-like protein containing a C-terminal caspase recruitment domain; CRP, C-reactive protein; PGE_2_, prostaglandin E_2_.

## 4 Therapeutic effects and mechanisms of plant metabolites in endometritis

**FIGURE 1:**
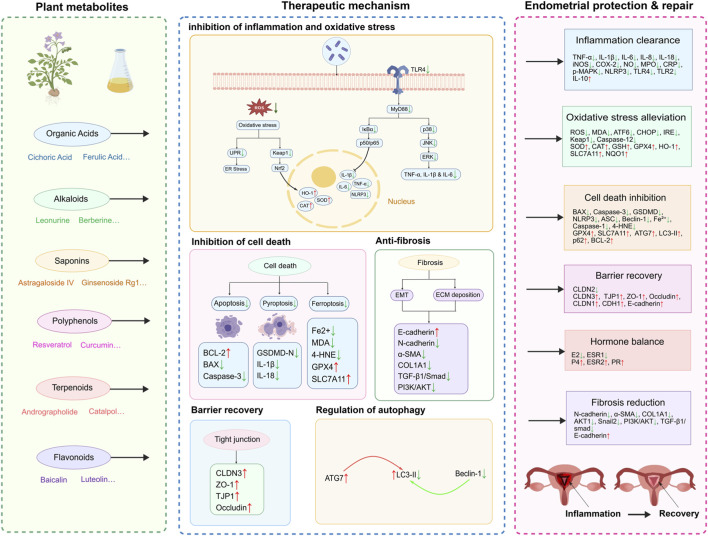
Schematic overview of the therapeutic mechanisms of plant metabolites in endometritis. Plant metabolites (left panel) exert therapeutic effects through multiple interconnected mechanisms (middle panel) that collectively promote endometrial protection and repair (right panel). The left panel presents representative plant metabolites from six major classes—organic acids, alkaloids, saponins, polyphenols, terpenoids, and flavonoids—which target diverse signaling pathways and cellular processes to alleviate endometritis. The middle panel illustrates the key therapeutic mechanisms through which these metabolites act. Based on representative examples such as berberine and astragaloside IV, plant metabolites inhibit inflammatory responses by suppressing the TLR4/MyD88/NF-κB and MAPK (JNK, ERK, p38) signaling cascades, thereby reducing the production of pro-inflammatory cytokines (TNF-α, IL-1β, IL-6). Concurrently, they activate the Keap1/Nrf2/HO-1 antioxidant pathway to upregulate the expression of HO-1, SOD, and CAT, suppress ROS generation and oxidative stress, and alleviate ER stress via inhibition of the UPR. In addition, plant metabolites regulate multiple forms of programmed cell death, including apoptosis (via modulation of BCL-2/BAX and caspases), pyroptosis (via GSDMD), and ferroptosis (via the GPX4/SLC7A11 axis, reducing MDA and 4-HNE). Autophagy (involving ATG7 and LC3-II) is also modulated to maintain cellular homeostasis. Regarding fibrosis, metabolites such as ginsenoside Rg1 inhibit EMT by regulating E-cadherin, N-cadherin, α-SMA, and COL1A1, and suppress the TGF-β1/Smad and PI3K/AKT pathways, thereby reducing ECM deposition and fibrosis. Furthermore, plant metabolites restore endometrial barrier integrity by regulating tight junction proteins, including CLDN3, TJP1, and ZO-1, while autophagy is modulated to balance cellular repair and survival. The right panel summarizes the integrated outcomes of these mechanisms, which collectively lead to inflammation clearance, oxidative stress alleviation, cell death inhibition, barrier recovery, hormonal balance restoration (modulation of ESR1, ESR2, PR, E2, P4), and fibrosis reduction. Collectively, these actions result in endometrial tissue protection, functional restoration, and improved reproductive outcomes. Abbreviations: TLR4, Toll-like receptor 4; MyD88, myeloid differentiation primary response 88; NF-κB, nuclear factor kappa-B; MAPK, mitogen-activated protein kinase; JNK, c-Jun N-terminal kinase; ERK, extracellular signal-regulated kinase; TNF-α, tumor necrosis factor-α; IL-1β, interleukin-1β; IL-6, interleukin-6; ROS, reactive oxygen species; Keap1, kelch-like ECH-associated protein 1; Nrf2, nuclear factor erythroid 2-related factor 2; HO-1, heme oxygenase-1; SOD, superoxide dismutase; CAT, catalase; ER, endoplasmic reticulum; UPR, unfolded protein response; ATF6, activating transcription factor 6; CHOP, C/EBP homologous protein; IRE, inositol-requiring enzyme; MPO, myeloperoxidase; NO, nitric oxide; CRP, C-reactive protein; BCL-2, B-cell lymphoma-2; BAX, Bcl-2-associated X protein; GSDMD, gasdermin D; GPX4, glutathione peroxidase 4; SLC7A11, solute carrier family 7 member 11; MDA, malondialdehyde; 4-HNE, 4-hydroxynonenal; ATG7, autophagy-related protein 7; LC3-II, microtubule-associated protein 1 light chain 3-II; EMT, epithelial-mesenchymal transition; α-SMA, α-smooth muscle actin; COL1A1, collagen type I alpha 1 chain; TGF-β1, transforming growth factor-β1; Smad, small mothers against decapentaplegic; PI3K, phosphatidylinositol 3-kinase; AKT, protein kinase B; CLDN, claudin; TJP1, tight junction protein 1; ZO-1, zonula occludens-1; ECM, extracellular matrix; ESR1, estrogen receptor α; ESR2, estrogen receptor β; PR, progesterone receptor; E2, estradiol; P4, progesterone.

Plant metabolites, including organic acids, alkaloids, saponins, polyphenols, terpenoids, and flavonoids, exert therapeutic effects against endometritis through distinct yet often overlapping mechanisms. These primarily involve the suppression of inflammatory responses, alleviation of oxidative stress, enhancement of endometrial cell activity, fortification of the endometrial barrier, regulation of hormonal balance, and inhibition of fibrosis. Figure 1 provides a schematic overview of these mechanisms, summarizing the key signaling pathways and cellular processes targeted by plant metabolites in endometritis.

### 4.1 Suppression of endometrial inflammatory responses

The anti-inflammatory action of plant metabolites is primarily achieved through the modulation of key signaling cascades that drive the pathological inflammation characteristic of endometritis.

#### 4.1.1 Inhibition of the NF-κB signaling pathway

The NF-κB signaling pathway is a central regulator of inflammatory responses (Guo et al., 2024) and is critically involved in the pathogenesis of endometritis, making it a prime therapeutic target (Zdrojkowski et al., 2023). Upon activation by stimuli (such as LPS), NF-κB translocates to the nucleus to drive the expression of pro-inflammatory cytokines (e.g., TNF-α, IL-6, IL-1β). Numerous plant metabolites have been shown to ameliorate endometritis by inhibiting various steps of this pathway. For instance, metabolites such as cichoric acid (Wang et al., 2022), ferulic acid (Yin et al., 2019), berberine (Fu et al., 2015), astragaloside IV (Wang et al., 2019), and puerarin (Kim et al., 2022) can reduce IκBα phosphorylation and degradation, thereby blocking NF-κB activation and subsequent pro-inflammatory cytokine release. Others, like alpinetin (Liang et al., 2018), chlorogenic acid (Gao et al., 2021a), and magnolol (Luo et al., 2013), intervene NF-κB activation and cytokine release by downregulating the LPS receptor TLR4 or its downstream adaptors, as seen with engeletin (Wu et al., 2016). Resveratrol offers an alternative mechanism by activating Sirt1, which in turn inhibits NF-κB signaling (Han et al., 2021).

#### 4.1.2 Inhibition of mitogen-activated protein kinase (MAPK) signaling pathways

The MAPK signaling pathways, including extracellular signal-regulated kinase (ERK), p38, and c-Jun N-terminal kinase (JNK), are crucial transducers of inflammatory signals in endometritis. Their activation leads to the phosphorylation of transcription factors such as activator protein one and the subsequent upregulation of pro-inflammatory genes (Pelech and Sanghera, 1992; Weiss et al., 2003; Vervaeke and Lamkanfi, 2025). Several plant metabolites target this axis. It has been demonstrated that nuciferine (Kim et al., 2022), ferulic acid (Yin et al., 2019), punicalagin (Lyu et al., 2017) and piperine (Zhai et al., 2016) inhibit the phosphorylation of ERK, JNK, and p38, downregulating the expression of IL-1β, IL-6, TNF-α, and IL-8. Astragaloside IV and baicalin also directly inhibit JNK phosphorylation and activator protein one activation, mitigating endometrial inflammation (Wang et al., 2019; Yang D. et al., 2022).

#### 4.1.3 Inhibition of NOD-Like receptor pyrin domain-containing 3 (NLRP3) inflammasome activation

The NLRP3 inflammasome plays a pivotal role in amplifying inflammatory responses in endometritis. Its overactivation leads to the maturation and secretion of potent pro-inflammatory cytokines like IL-1β and IL-18, exacerbating tissue injury (Li L. et al., 2023; Yang et al., 2023; Zou et al., 2025). Plant metabolites can inhibit this pathway at multiple levels. Ginsenoside Rg1 reduces reactive oxygen species (ROS) generation, which inhibits NLRP3 inflammasome activation, downregulates the expression of ASC (apoptosis-associated speck-like protein containing a C-terminal caspase recruitment domain), caspase-1, and IL-1β (Song et al., 2023). Similarly, puerarin inhibits the P2X7/NLRP3 pathway activation (Yuan et al., 2024), while rosmarinic acid and timosaponin AIII directly suppress the expression of NLRP3 complex components, reducing downstream inflammatory damage (Taravat et al., 2023; Jiang and Xu, 2024).

#### 4.1.4 Reduction of inflammatory cell infiltration

The cumulative effect of inhibiting these pathways is a reduction in the recruitment and activity of immune cells. Excessive infiltration of immune cells, particularly neutrophils, into the endometrium is a hallmark of endometritis and contributes to tissue damage (Zeng S. et al., 2022; Li H. et al., 2023). Plant metabolites can mitigate this pathology by modulating key markers and mediators of leukocyte recruitment and activation. For example, certain metabolites reduce myeloperoxidase activity, diminish neutrophil infiltration, and lower C-reactive protein levels, thereby mitigating uterine tissue damage (Wu et al., 2017; Gao et al., 2021a; Wang et al., 2022). Others reduce nitric oxide levels in uterine tissue, attenuating vasodilation and vascular permeability to curb inflammatory cell infiltration (Fu et al., 2015; Fu et al., 2021a). Furthermore, resveratrol can inhibit the expression of chemokines such as CINC-3 (cytokine-induced neutrophil chemoattractant-3), IL-6, LIX (LPS-induced CXC chemokine), and CINC-1/CXCL-1 (C-X-C motif chemokine ligand-1), reducing neutrophil recruitment to the endometrium and lessening inflammatory damage (Han et al., 2021).

### 4.2 Alleviation of endometrial oxidative stress

Oxidative stress and inflammation form a vicious cycle that exacerbates endometrial damage. During inflammation, activated immune cells generate ROS. An overwhelmed antioxidant defense system leads to oxidative damage to cellular components, which in turn activates pro-inflammatory pathways like NF-κB, recruiting more immune cells and perpetuating inflammation (Wang et al., 2025). Plant metabolites can break this cycle through both direct and indirect antioxidant mechanisms.

#### 4.2.1 Activation of the Nrf2 signaling pathway

The Nrf2 pathway is a central cellular defense mechanism against oxidative stress. Under oxidative conditions, Nrf2 dissociates from its inhibitor Keap1, translocates to the nucleus, and activates the expression of antioxidant genes like heme oxygenase-1 (HO-1) and NQO1 (NAD(P)H Dehydrogenase, Quinone 1). This not only combats oxidative stress but can also suppress NF-κB-mediated inflammation (Saha et al., 2020; Tonolo et al., 2022). Metabolites such as thymol (Wu et al., 2017), fisetin (Jiang et al., 2021b), and hydroxytyrosol (Gugliandolo et al., 2020) can directly scavenge ROS. Concurrently, metabolites like cichoric acid (Wang et al., 2022), luteolin (Gao et al., 2024), saikosaponin A (Wei et al., 2016), and icariin (Shaukat et al., 2022) activate the Nrf2/HO-1 pathway, enhancing the cellular antioxidant capacity. Others, such as berberine (Fu et al., 2015), chlorogenic acid (Gao et al., 2021a), and tanshinone IIA (Fu et al., 2021b) promote Nrf2 nuclear translocation by downregulating its inhibitor Keap1. Collectively, these actions reduce malondialdehyde accumulation, restore glutathione levels, and boost the activity of enzymes like superoxide dismutase and catalase, thereby alleviating lipid peroxidation and bolstering endometrial resilience.

#### 4.2.2 Inhibition of endoplasmic reticulum stress

Inflammation-induced ROS can disrupt calcium homeostasis and cause misfolded protein accumulation, activating the unfolded protein response and endoplasmic reticulum stress (ERS). While initially protective, persistent ERS can trigger cell death and further inflammatory signaling through pathways like IRE1α-XBP1, forming a positive feedback loop with inflammation (Demine et al., 2020; Fionda and Sciume, 2024). LPS activates ERS in endometrial epithelial cells, increasing the expression of related proteins (ATF6, CHOP, IRE1, and cleaved caspase-12). Honokiol can dose-dependently reduce these protein levels, inhibiting ERS (Chen et al., 2021) and endometrial cell apoptosis. Naringin exerts its therapeutic effect by regulating the ERS-PI3K/AKT-autophagy axis (Lu et al., 2024), suppressing the unfolded protein response and shifting the cytokine profile from pro-inflammatory (IL-6, IL-1β, TNF-α) to anti-inflammatory (IL-10).

### 4.3 Enhancement of endometrial cellular vitality

By mitigating inflammation and oxidative stress, plant metabolites also modulate various forms of programmed cell death, preserving endometrial cellular function and promoting tissue integrity.

#### 4.3.1 Inhibition of apoptosis, pyroptosis and ferroptosis

In endometritis, sustained inflammation and oxidative stress trigger multiple cell death pathways. Pro-apoptotic proteins (Bax, cleaved caspase-3) are upregulated, while the anti-apoptotic Bcl-2 is downregulated in endometrial epithelial cells. Concurrently, GSDMD (gasdermin D)-mediated pyroptosis and GPX4-regulated ferroptosis are induced. Honokiol can dose-dependently reverse the apoptotic imbalance (Chen et al., 2021), while rosmarinic acid can reduce GSDMD expression to inhibit endometrial cell pyroptosis (Taravat et al., 2023). Regarding ferroptosis, metabolites like cichoric acid (Wang et al., 2022), nuciferine (Kim et al., 2022), and puerarin (Wei et al., 2016) reduce intracellular ferrous iron (Fe^2+^) and lipid peroxidation products (malondialdehyde, 4-hydroxynonenal), while increasing the expression of the key ferroptosis regulator GPX4 and its co-factor SLC7A11, thereby reducing iron deposition and ferroptosis.

#### 4.3.2 Modulation of cellular autophagy

Autophagy plays a dual role in cellular survival. Appropriate autophagy aids in cellular repair and survival, whereas excessive activation can lead to cell death (Liu S. et al., 2023). In the context of endometritis, baicalin activates autophagy, increasing levels of autophagy-related proteins like ATG7 (autophagy-related protein 7) and LC3-II, which helps clear damaged organelles in endometrial cells (Yang D. et al., 2022). Interestingly, by modulating autophagy, baicalin promotes the redistribution of tight junction proteins claudin-3 (CLDN3) and tight junction protein 1 (TJP1) from the cytoplasm to the cell membrane and degrades membrane-bound claudin-2 (CLDN2), thereby contributing to the restoration of endometrial barrier function (Yang D. et al., 2022). Conversely, naringin can dose-dependently suppress excessive autophagy in endometrial cells during endometritis, as evidenced by a decreased LC3-II/LC3-I ratio and Beclin-1 levels, alongside increased p62 levels, thereby attenuating autophagy-associated cell death (Lu et al., 2024). This highlights the need for context-dependent modulation of this pathway.

### 4.4 Strengthening of the endometrial barrier function

The endometrial barrier, primarily constituted by tight junctions between epithelial cells, is essential for maintaining uterine homeostasis. Pathogens like *E. coli*, *Staphylococcus* spp., and LPS can disrupt this barrier to invade the endometrium, excessively activating the immune system and causing inflammatory damage. Some plant metabolites can reinforce the endometrial barrier to prevent and mitigate endometrial inflammation.

Tight junctions comprise transmembrane proteins and adaptor/scaffold proteins. CLDN1 and CLDN3 promote epithelial tightness, whereas CLDN2 is a pore-forming claudin that reduces tightness (Lu et al., 2022). In LPS-induced endometritis, the mRNA expression of tight junction proteins like claudins, CDH1 (cadherin 1), and TJP1 is significantly reduced. Baicalin (Yang D. et al., 2022) and hydroxytyrosol (Gugliandolo et al., 2020) can dose-dependently increase the levels of CLDN3 and TJP1 in endometrial epithelial cells while decreasing CLDN2 expression, facilitating barrier maintenance. In *E. coli*-induced endometritis, timosaponin AIII can significantly increase ZO-1 (zonula occludens-1), occludin, and CLDN1 expression, promoting tight junction formation (Wei et al., 2016). Beyond expression levels, proper protein localization is critical. In endometritis, LPS causes abnormal cytoplasmic aggregation of CLDN3 and TJP1 in endometrial epithelial cells, hindering intercellular tight junction formation (Lu et al., 2022). Baicalin facilitates the redistribution of these proteins from the cytoplasm to the cell membrane, mediated by LC3-II-positive structures, while reducing membrane localization of CLDN2 (Yang D. et al., 2022). This promotes proper tight junction assembly, maintains normal barrier function, and alleviates endometritis.

### 4.5 Regulation of endometrial hormonal balance

Endometritis is often associated with hormonal disturbances, typically characterized by elevated serum estradiol and decreased progesterone levels (Sheldon et al., 2002). By restoring this balance, plant metabolites can create a more favorable environment for endometrial repair.

In *E. coli*-induced rat endometritis, resveratrol significantly lowers serum estradiol and raises progesterone levels, showing comparable efficacy to the traditional combination therapy of prostaglandin F2α and marbofloxacin (Han et al., 2021). Increased progesterone helps suppress excessive inflammation, while decreased estradiol reduces abnormal endometrial stimulation, thereby creating a favorable environment for repair (Raghupathy and Szekeres-Bartho, 2022).

Hormone receptors are vital for endometrial function (Jia et al., 2015). Endometritis downregulates estrogen receptor β (*ESR2*) gene expression. Resveratrol significantly upregulates *ESR2* gene expression in uterine tissue while downregulating *ESR1* (Han et al., 2021). Furthermore, resveratrol enhances estrogen receptor protein expression in the uterine stroma and, when combined with marbofloxacin, increases estrogen receptor expression in uterine glands (Han et al., 2021). High ESR2 expression aids in maintaining endometrial homeostasis and cellular regeneration, while downregulated ESR1 may reduce excessive proliferation and promote inflammatory resolution (Gibson et al., 2020). Normal progesterone receptor expression is essential for endometrial responsiveness to progesterone (Piette, 2018). In endometritis, resveratrol alone does not significantly affect progesterone receptor expression, but its combination with marbofloxacin significantly increases progesterone receptor protein expression in the uterine epithelium (Han et al., 2021). This enhances endometrial sensitivity to progesterone, potentially amplifying its anti-inflammatory effects to inhibit pro-inflammatory factor release and reduce inflammatory damage.

The hormonal modulatory effects of plant metabolites like resveratrol are intriguing but primarily observed in animal models. A significant translational challenge lies in understanding the systemic and local endocrine effects of these metabolites in women, whose hormonal cycles are more complex. Long-term safety and the impact on the hypothalamic-pituitary-ovarian axis must be thoroughly evaluated before considering them for clinical use, especially in women of reproductive age.

### 4.6 Anti-fibrotic effects

If inflammation persists, it can lead to endometrial fibrosis, where normal tissue is replaced by fibrous tissue, leading to dysfunction. A key process in this pathology is epithelial-mesenchymal transition (EMT), where epithelial cells acquire mesenchymal characteristics (Sun et al., 2024). Ginsenoside Rg1 can dose-dependently inhibit the EMT process by upregulating the epithelial marker E-cadherin and downregulating the mesenchymal markers N-cadherin and α-smooth muscle actin (Song et al., 2023). Concurrently, ginsenoside Rg1 significantly reduces LPS-induced ROS levels and increases glutathione, similar to the ROS inhibitor N-acetylcysteine, thereby inhibiting fibrosis through alleviating oxidative stress (Song et al., 2023). Tanshinone IIA can inhibit the NF-κB/Snail2 signaling pathway, similarly suppressing LPS-induced EMT and alleviating fibrosis (Fu et al., 2021b).

The PI3K-AKT pathway, involved in cell proliferation and fibrosis, is also a target (Huang et al., 2025). In LPS-induced endometritis, this pathway is activated, upregulating the gene expression of type I collagen α1 chain and AKT1, promoting uterine tissue fibrosis (Jiang et al., 2021a). Leonurine inhibits PI3K-AKT pathway activation, significantly reducing type I collagen α1 and AKT1 expression, thereby attenuating inflammation-induced endometrial fibrosis and preserving uterine structural integrity (Shao et al., 2024).

## 5 Limitations and future perspectives

Despite the promising therapeutic potential of plant metabolites in endometritis, several critical limitations must be addressed to bridge the gap between bench-side discoveries and clinical applications.

### 5.1 Limitations in experimental models

Substantial limitations exist in the experimental models used to evaluate plant metabolites for endometritis. First, the majority of studies rely on acute LPS-induced models, which may not adequately capture the complex, polymicrobial, and often chronic nature of clinical endometritis. Second, most evidence is derived from cell cultures *in vitro* or small animal models, with a notable lack of well-designed studies in large animal models that better recapitulate human reproductive physiology. Third, existing studies employ diverse models (e.g., LPS vs. live bacteria; mice vs. rats vs. cows), different treatment durations, and a wide range of metabolite concentrations, making it difficult to synthesize findings or rank the therapeutic potential of different metabolites. The lack of standardized, clinically relevant models of chronic and recurrent endometritis is particularly problematic for evaluating anti-fibrotic and tissue-repairing effects.

### 5.2 Gaps in mechanistic understanding

Significant gaps remain in mechanistic understanding. While many plant metabolites have been shown to modulate canonical pathways such as NF-κB, the precise molecular targets—including direct binding proteins or upstream sensors—remain unidentified for most plant metabolites. The interplay between different signaling pathways (e.g., crosstalk between inflammation and ferroptosis) and the temporal dynamics of their activation during disease progression are poorly characterized. Comparative studies on the efficacy, potency, and potential synergies or antagonisms among different metabolites targeting the same pathway are scarce.

Research on oxidative stress mitigation by plant metabolites has predominantly focused on the Nrf2 pathway, while the interplay between different antioxidant systems and their relative contribution to endometrial repair remains to be clarified. The dual role of ROS as both damaging molecules and essential signaling mediators in the context of endometritis necessitates precise, context-dependent modulation by therapeutic agents, yet such modulation has not been systematically explored in plant metabolite studies.

Regarding cell death regulation, the optimal timing and context for modulating apoptosis, pyroptosis, ferroptosis, and autophagy by plant metabolites to promote endometrial cell survival without compromising pathogen clearance remain undefined. Furthermore, the causal links between the inhibition of specific cell death modalities by these metabolites and the improvement of concrete functional outcomes, such as embryo implantation and pregnancy maintenance, have not been established.

For barrier function, current studies on plant metabolites have primarily focused on static protein expression levels of tight junction components, while the dynamic regulation of the endometrial barrier—including rapid cytoskeletal reorganization and complex interactions with the local microbiota and immune cells—remains poorly understood. In fibrosis research, most studies have only examined the effects of plant metabolites during acute or subacute inflammation, leaving the question of whether these metabolites can reverse established fibrosis unanswered. The relative contribution of inhibiting EMT versus directly targeting activated fibroblasts or collagen cross-linking also remains unclear.

### 5.3 Challenges in clinical translation

The field suffers from a paucity of rigorous clinical validation. To date, no randomized controlled trials have been conducted in human patients. Critical parameters—including optimal dosage, route of administration, bioavailability at the target tissue, and long-term safety profiles—remain largely unexplored. Moreover, the potential for metabolite toxicity at therapeutic doses and the impact on fertility outcomes have not been systematically evaluated.

### 5.4 Future research priorities

To overcome these challenges and accelerate clinical translation, future research should prioritize the following directions:

- Elucidating precise molecular targets and signaling crosstalk. Advanced tools such as chemical proteomics, CRISPR-based genetic screens, and systems biology approaches should be employed to map the complex interactome of plant metabolites and elucidate the temporal dynamics of pathway activation.
- Exploring rational combination strategies. Both synergies among different plant metabolite classes and adjunctive use with conventional antibiotics should be investigated to enhance efficacy, reduce required doses, and potentially mitigate antimicrobial resistance.
- Advancing mechanistic understanding of key therapeutic processes. Future studies should explore the interplay between different antioxidant systems beyond the Nrf2 pathway, define the optimal timing for modulating cell death pathways, establish causal links between cell death inhibition and functional outcomes, investigate dynamic regulation of the endometrial barrier, and evaluate the potential of plant metabolites to reverse established fibrosis.
- Validating efficacy in more physiologically relevant models. Future studies should utilize chronic endometritis models, endometrial organoid systems that recapitulate human tissue architecture, and large animal models (e.g., bovine or porcine) that more closely mirror human reproductive pathophysiology.
- Conducting rigorous preclinical toxicology and pharmacokinetic studies. Systematic evaluation of safety margins, tissue distribution, and metabolic fate is needed, followed by well-designed early-phase clinical trials in patients with endometritis, with particular attention to reproductive outcomes and long-term safety.

## 6 Conclusion

In conclusion, this review provides a comprehensive and critical synthesis of the therapeutic potential of plant metabolites in endometritis, with a particular focus on critically assessing the evidence and identifying key gaps in the current literature. The findings highlight the multifaceted mechanisms of plant metabolites, including the suppression of inflammation, alleviation of oxidative stress, regulation of cell death, restoration of barrier integrity, modulation of hormonal balance, and inhibition of fibrosis, which offer a distinct advantage over conventional antibiotic therapy that primarily targets pathogens. However, significant challenges remain. The predominance of acute LPS-induced models limits the translational relevance of current findings, the precise molecular targets of most plant metabolites remain unidentified, and rigorous clinical validation is entirely lacking. Addressing these gaps through systematic translational research, including the development of physiologically relevant models, elucidation of molecular targets, and well-designed clinical trials, will be essential to advance the field. Ultimately, plant metabolites may provide safer, more effective alternatives or adjuncts to conventional antibiotics, improving reproductive health outcomes for women worldwide.
